# Current European landscape of in-hospital cardiac arrest registries mapped by a cross-sectional survey including barriers and enablers setting up a registry

**DOI:** 10.1016/j.resplu.2026.101533

**Published:** 2026-10-16

**Authors:** Christoph Veigl, Jacqueline Eleonora Ek, Gavin D. Perkins

**Affiliations:** a Department of Anesthesiology and Intensive Care Medicine, University Hospital St. Pölten – NOE LGA, Karl Landsteiner University, St. Pölten, Austria; b PULS – Austrian Cardiac Arrest Awareness Association, Vienna, Austria; c Department of Medicine, Mater Dei Hospital, Malta; d Warwick Medical School, University of Warwick, Coventry, England, United Kingdom

**Keywords:** In-hospital cardiac arrest, Registry, Resuscitation, Cardiopulmonary resuscitation, CPR, Cardiac Arrest

## Abstract

**Background:**

In-hospital cardiac arrest (IHCA) has a high mortality rate. To measure and improve resuscitation outcomes, data collection through registries plays a key role. This study aims to evaluate the current landscape of IHCA registries across national resuscitation councils affiliated with the European Resuscitation Council.

**Methods:**

A cross-sectional survey was distributed among national resuscitation councils collecting data on the presence and type of IHCA registries, registry coverage and data variables collected. Findings informed an expert discussion on barriers and enablers establishing such registries and potential next steps.

**Results:**

30/34 (88%) national resuscitation councils replied to the survey. Seven have no IHCA registry (23%), 11 a local registry (37%), two a local and regional registry (7%), five a national registry (17%), and two countries participate in a national registry in another country (7%). Of the included registries 17 (94%) are collecting cardiac arrests in adults, 13 (72%) in children, and four (22%) in neonates. Most registries are collecting location of cardiac arrest (95%), the initial rhythm (95%), and if return of spontaneous circulation is achieved (100%), while long term neurological outcome is only collected by four registries (21%). Overall, the AI-assisted descriptive content analysis demonstrated broad consensus regarding the value of registries, while highlighting substantial logistical, legal, and financial barriers.

**Conclusion:**

Our survey demonstrates considerable variation in the existence, population coverage, and data collection practices of IHCA registries. While core cardiac arrest variables are collected by most registries, data on long-term outcomes remain limited.

## Graphical abstract

**Figure:**
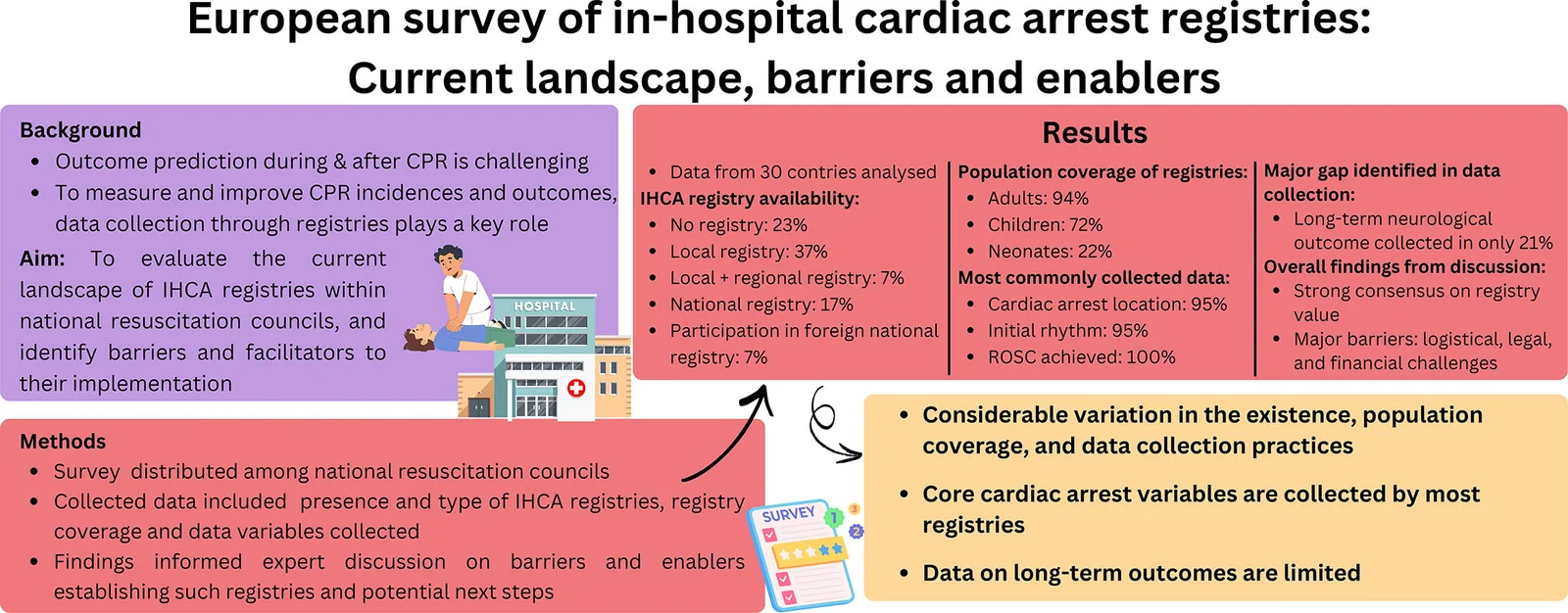

## Introduction

In-hospital cardiac arrest (IHCA) represents a major challenge for healthcare systems with an incidence of 0.7–2.8 per 1000 admissions and high mortality rates.^1^, ^2^, ^3^, ^4^, ^5^, ^6^ Through standardised data collection, registries play a central role in quality measurement and provide an objective framework to evaluate opportunities for improvement and guide practices that can be shared across institutions and countries.^3^, ^7^, ^8^ The 2019 updated Utstein-style reporting templates provides a structured framework to collect data in an IHCA registry to compare systems of care for cardiac arrest.^9^

Beyond benchmarking, registry systems could contribute directly improving quality of care and patient outcomes.^10^, ^11^ By analysing key variables such as response times, treatment interventions, and survival outcomes, registries allow healthcare systems to identify modifiable factors associated with better outcomes.^11^ In addition, IHCA registries generate evidence to inform healthcare policy, increase awareness of IHCA, and the development of standardised protocols and guidelines. Historically, out-of-hospital cardiac arrest (OHCA) has received greater public and scientific attention, while IHCA has often remained underrepresented despite its significant similar burden.^12^, ^13^, ^14^, ^15^

This study aimed to evaluate the current landscape of IHCA registries within national resuscitation councils affiliated with the European Resuscitation Council, and identify barriers and facilitators to their implementation.

## Methods

The European Resuscitation Council is a network of national resuscitation councils, predominantly in Europe, who work collaboratively to preserve human life by making high quality resuscitation available to all.

The survey was conducted to inform an in-person discussion about the current status on IHCA registries across national resuscitation council countries among representatives of these councils which was held in Vienna on 7 March. The checklist for reporting results of internet e-surveys (CHERRIES statement) was used to report the methods and results.^16^

Participation in the survey was voluntary, no personal data were collected, no formal ethical approval was required, and no incentives were offered. The survey using the electronic survey tool Microsoft Forms (Microsoft Corporation, Redmond, WA, USA) was distributed among the 34 national resuscitation council chairs and vice-chairs on 3 February 2026 via e-mail, after being tested by one Young ERC committee member. A reminder was sent to representatives of countries without a response after 7 and 14 days. Only one response per national resuscitation council was required. In cases where multiple responses were received from the same country, discrepancies were resolved through discussion among respondents. The survey (Supplement A) comprised four multiple-choice and five short-answer questions listed on one page. These include country of response, number of hospitals, proportion of hospitals in the country covered by IHCA registries and basic information about existing IHCA registries. Only the country of response was a mandatory field. For the purpose of this survey, a local registry was defined as a registry systematically collecting cardiac arrest data from a single hospital, whereas a regional registry was defined as a registry collecting cardiac arrest data from multiple hospitals within a defined geographical region (e.g., a state or province). National registries were defined as registries covering multiple regions or the entire country.

All national resuscitation council chairs and vice-chairs were invited to participate at the in-person event, and participants were divided into four small discussion groups, each consisting of approximately 10 participants and one expert moderator taking notes. The groups addressed the importance of an IHCA registry, barriers and enablers to establish such registries, the type of support that should be provided by national resuscitation councils and the ERC, and potential next steps.

Quantitative data were summarised using descriptive statistics, with categorical variables reported as counts and percentages. Qualitative data generated from the group discussions were analysed with support by artificial intelligence (AI) (Open-AI GPT5.3): notes were uploaded, GPT5.3 was asked to code key points into recurring themes, and to summarise similar responses across the discussion groups to identify common patterns based on the frequency for every question. The used prompts can be found in Supplement A. Afterwards, the identified patterns were manually reviewed by one investigator (*CV*) to confirm that they were supported by the discussion notes.

## Results

After de-duplication, responses from 30 out of the 34 contacted countries (88%) were analysed. The included countries comprise a population of approximately 775 million people and approximately 23,688 hospitals.

Of the included countries seven have no active IHCA registry (23%) (of those one had previously a regional registry, which was no longer active at the time of the survey), eleven a local registry (37%), two a local and regional registry (7%), five a national registry (17%), two countries participate in a national registry in another country (7%), one a national and regional registry (3%), one currently in the pilot phase of a national registry (3%) and in one country the government is collecting data, but it is not publicly available (3%) (Fig. 1 and Table 1). Data submitted from the country with the terminated registry was included in the following analysis.

**Fig. 1:**
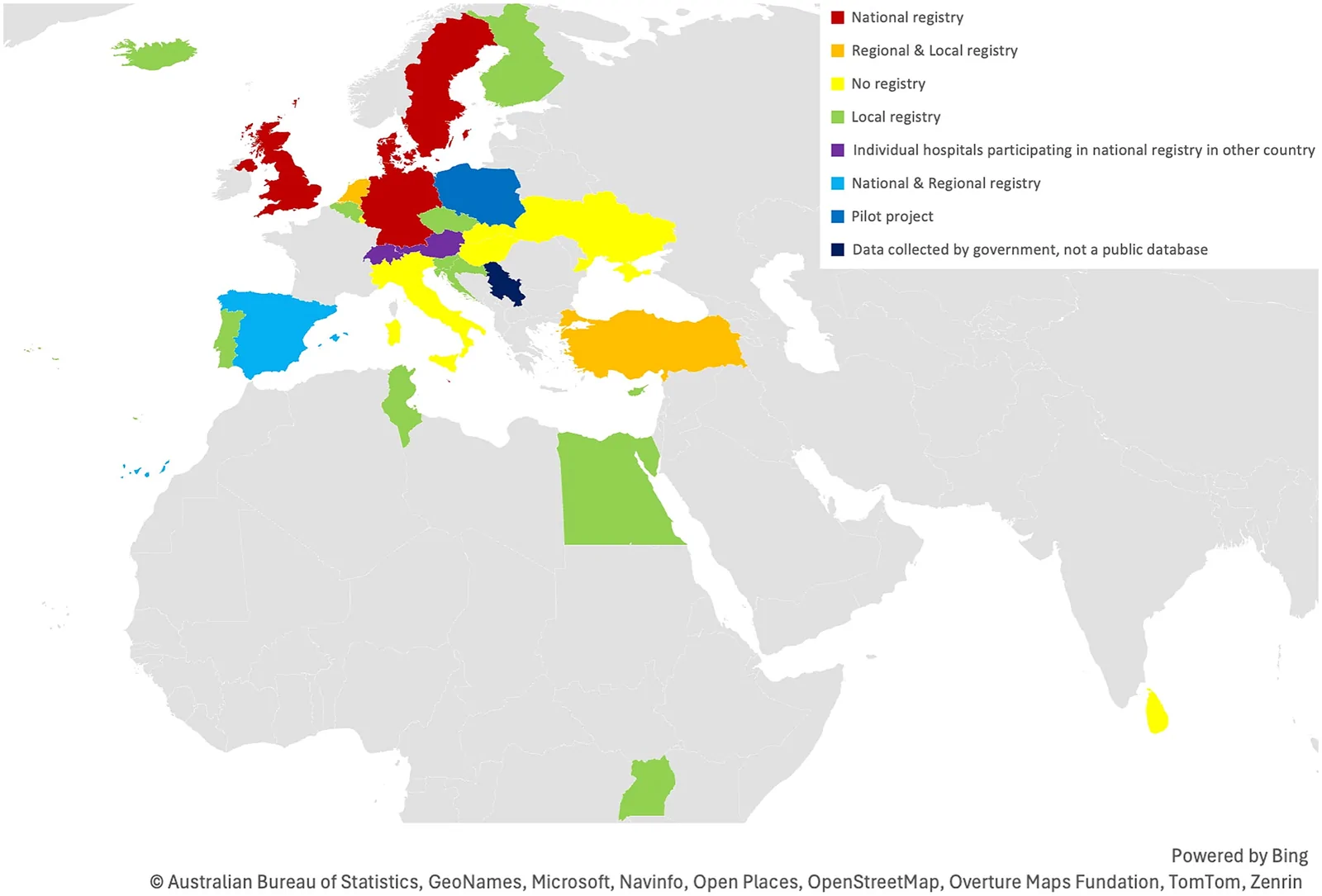
**Geographic overview of different in-hospital cardiac arrest registries identified in our survey**.

**Table 1:** Overview of collected IHCA registries.

No active registry, *n* (%)	7 (23)
– Terminated regional registry, *n* (%)	1 (3)
Local registry, *n* (%)	11 (37)
Regional & local registry *n* (%)	2 (7)
National registry, *n* (%)	5 (17)
National & regional registry, *n* (%)	1 (3)
Individual hospitals participating in national registry in other country, *n* (%)	2 (7)
Pilot phase of a national registry, *n* (%)	1 (3)
Government is collecting data, but not publicly available, *n* (%)	1 (3)

The mean proportion of covered hospitals is 48% (SD: 42%), with four registries (17%) covering the full population. Of the included registries 17 (94%) are collecting cardiac arrests in adults, 13 (72%) in children, four (22%) in neonates, and for six registries (25%) there is no further information about covered population. Nine IHCA registries (45%) are collecting cardiac arrest data for all cardiac arrests occurring in the hospital, another nine (45%) for cardiac arrests attended by a resuscitation team, three (15%) for cardiac arrests occurring in a particular area of the hospital (two only in the emergency department (ED) and one in the ED and operating theatre).

For five countries (21%) there was no information about the collected data, and following data is collected by registries of the remaining nineteen countries: In sixteen (84%) incidences of IHCA is collected, sixteen (84%) patient demographics, eighteen (95%) location of cardiac arrest in the hospital, eighteen (95%) the initial rhythm, nineteen (100%) if return of spontaneous circulation (ROSC) is achieved, fourteen (74%) survival to discharge/30 days, nine (47%) neurological outcome at discharge/30 days, seven (37%) long term survival (>30 days) and in four (21%) long term neurological outcome (>30 days). A more detailed overview of the collected information can be found in Table 2 and Supplement B*.*

**Table 2:** Results of survey grouped according to the registry type in the country.

	**All** **(*n* = 24)**^a^	**Local registry** **(*n* = 11)**	**Regional & local registry** **(*n* = 3)**^a^	**National registry** **(*n* = 10)**^b^
Proportion of hospitals in the country covered by registries, % (SD)	42 (48)	32 (46)	100 (N/A)	56 (40)
– No further information, *n* (%)	7 (30)	5 (46)	2 (67)	0 (0)
**Population covered by registries:**^c^				
– No further information, *n* (%)	6 (25)	4 (37)	1 (33)	1 (10)
– Adults, *n* (%)^d^	17 (94)	6 (86)	2 (100)	9 (100)
– Children, *n* (%)^d^	13 (72)	4 (57)	2 (100)	7 (78)
– Neonates, *n* (%)^d^	4 (22)	1 (14)	1 (50)	2 (22)
**Cardiac arrests included in the registries:**^f^				
– No further information, *n* (%)^d^	4 (17)	3 (28)	1 (33)	0 (0)
– All cardiac arrest patients occurring, *n* (%)^d^	9 (45)	3 (38)	0 (0)	6 (60)
– Cardiac arrests attended by resuscitation team, *n* (%)^d^	9 (45)	3 (38)	2 (100)	4 (40)
– Cardiac arrests occurring in particular part of the hospital, *n* (%)^d^	3 (15)	2 (25)	0 (0)	0 (0)
◦ Emergency department^g^	3 (100)	2 (100)	0 (0)	0 (0)
◦ Operation room^g^	1 (33)	0 (0)	0 (0)	0 (0)
**Data collected by registries:**^c^				
– No information about collected data, *n* (%)	5 (21)	3 (28)	1 (33)	1 (10)
– Incidence of IHCA (e.g. cardiac arrests per admission), *n* (%)^d^	16 (84)	6 (75)	2 (100)	8 (89)
– Patient demographics, *n* (%)^d^	16 (84)	8 (100)	2 (100)	6 (67)
– Hospital name, *n* (%)^d^	16 (84)	5 (63)	2 (100)	9 (100)
– Location of cardiac arrest in the hospital, *n* (%)^d^	18 (95)	7 (88)	2 (100)	9 (100)
– Initial rhythm, *n* (%)^d^	18 (95)	7 (88)	2 (100)	9 (100)
– ROSC achieved, *n* (%)^d^	19 (100)	8 (100)	2 (100)	9 (100)
– Survival to discharge/30 days, *n* (%)^d^	14 (74)	4 (50)	2 (100)	8 (89)
– Neurological outcome at discharge/30 days, *n* (%)^d^	9 (47)	2 (25)	1 (50)	6 (67)
– Long term survival (>30 days), *n* (%)^d^	7 (37)	2 (25)	0 (0)	5 (56)
– Long term neurological outcome (>30 days), *n* (%)^d^	4 (21)	1 (13)	0 (0)	3 (33)

N/A = Not applicable; IHCA = In-hospital cardiac arrest; ROSC = Return-of-spontaneous circulation.

a Including information about one regional registry, which is no longer active.

b Including information from countries participating in national registry of other country, information from country with national & regional registry, country in pilot phase of national country, and information from country, where government is collecting data, but it is not publicly available.

c Multiple answers possible.

d Percentages calculated from replies with further information.

f Only one answer.

g Percentages calculated from cardiac arrests occurring in particular part of the hospital.

Overall, the AI-assisted descriptive content analysis of notes during the in-person discussion demonstrated broad consensus regarding the value of IHCA registries while highlighting substantial logistical, legal, and financial barriers that require coordinated national and European action. Detailed information about the recurring themes in the group discussion can be found in Table 3.

**Table 3:** Overview about recurring themes in the group discussion during the NRC days.

Importance of an IHCA registry	– Quality measurement and benchmarking – Improvement of patient outcomes and survival – Evidence generation for policy, advocacy, and standardisation – Increased awareness of IHCA as a healthcare priority – Identification of training needs and healthcare gaps

Barriers to implementation	– Lack of funding and personnel resources – Complex data collection processes – Legal and regulatory concerns regarding data ownership, privacy, and consent – Coordination challenges between hospitals and institutions – Data quality and standardisation issues – Limited public or political awareness

Facilitators and enablers	– Easy-to-use and simplified data collection systems – Digital tools and automation, including artificial intelligence – Designated local coordinators – Political support and institutional willingness – Existing professional networks and shared expertise – Clear governance frameworks for data use

Recommended next steps for NRCs and/or ERC	– Conduct local assessments of existing infrastructure – Foster collaboration and knowledge-sharing between NRCs – Develop ERC leadership, including dedicated registry support roles – Seek European-level funding opportunities – Increase awareness through advocacy, survivors, and public engagement – Update and standardise IHCA data definitions and registry frameworks

ERC = European Resuscitation Council; IHCA = In-hospital cardiac arrest; NRC = National resuscitation council.

## Discussion

This overview of IHCA registries across national resuscitation council countries reveals substantial heterogeneity in their availability, scope, and data collection practices. While a majority of countries have established some form of registry, only a minority collect data in a national registry, and nearly one quarter lack any registry infrastructure. Over the past decade, there has been a marked expansion of OHCA registries, especially with a full population coverage. A recent survey within the EuReCa (European Registry of Cardiac Arrest) network reported that, among 27 European countries, only five countries (19%) had no OHCA registry in place and twenty-two (81%) had established an OHCA registry. Of these, nine (41%) achieved full population coverage, while 13 (59%) reported partial coverage.^17^ Even among existing IHCA registries in our survey, hospital coverage is highly variable and only 17% of the countries have full population coverage. The average inclusion is of less than half of the hospitals, indicating significant gaps in representativeness in these countries.

Data collection practices were noted to differ considerably. Although core variables such as ROSC and initial rhythm are widely reported, more comprehensive outcomes – especially long-term survival and neurological status are inconsistently collected. This variability could limit the comparability of data and the ability to inform recommendations. A consistently reported challenge is the lack of a universal definition of cardiac arrest, contributing to substantial variation in reported incidence rates of treated IHCA across Europe.^12^, ^13^, ^14^ The ERC therefore continues to recommend the adoption of the Utstein reporting guidelines for IHCA.^14^ These guidelines define incidence as the number of treated IHCAs per 1000 hospital admissions, excluding events occurring in the emergency department, while a cardiac arrest is defined by the delivery of chest compressions and/or defibrillation.^9^

You cannot improve what you do not measure. Despite differences in data collection, there is strong consensus during the discussion and among experts on the importance of IHCA registries for quality improvement, benchmarking, and research.^10^, ^12^, ^13^ The IHCA Utstein-style reporting template provides a structured guidance for collected data across 6 domains.^9^ While specific data to be collected should be adapted to local resources and can differ between individual institutions, to enable comparisons, adherence to Utstein definitions should be followed.^12^, ^13^, ^18^ For example, CPR training needs could be evaluated by measuring CPR quality,^19^ while measurement of time to defibrillation could provide insights for the need for easier and faster access to defibrillators.^20^ However, key barriers, including logistical challenges, legal constraints, and limited financial resources, continue to hinder their development.

One possible next step to collect data on IHCA could be a similar project to the EuReCa, an international, prospective, multi-centre registry study covering three months and focusing on the epidemiology, treatment, and outcomes of OHCA in Europe.^21^ Besides data gathering, such initiatives could help to raise awareness among stakeholders and build networks between different institutions.

## Limitations

Respondents were asked to answer at the national level, which may have limited data on regional variations and smaller, local registries. Although response options such as “partly” and open-end questions to explain regional differences were available, the survey primarily focused on national-level data. Responses may also have been subject to informant bias, as the survey was sent to NRC chairs and vice-chairs rather than registry custodians or data managers. Furthermore, as the survey was conducted in English – a non-native language for most participants – differences in interpretation of the questions may have influenced the responses. Another limitation is that the survey was only distributed among countries with an established national resuscitation council affiliated with the European Resuscitation Council indicating a degree of national co-ordination. Findings may differ from countries without a national council or one affiliated with other organisations. There was only one external reviewer testing the survey, before sending it out. Discussion notes were summarised with AI assistance and reviewed by a single investigator, without independent coding or assessment of inter-rater reliability. Finally, the study was based on self-reported data without independent verification. Despite these limitations, the survey highlights important differences in IHCA registries and identifies areas for future research.

## Conclusion

Our survey demonstrates considerable variation in the existence, population coverage, and data collection practices of IHCA registries across national councils affiliated with the European Resuscitation Council. While core cardiac arrest variables are collected by most registries, data on long-term outcomes remain limited. Despite these differences, there was broad consensus on the value and importance of IHCA registries.

## Declaration of AI and AI-assisted technologies in the writing process

During the preparation of this work the authors used OpenAI's Open-AI GPT5.3 to improve readability and language. After using this tool/service, the authors reviewed and edited the content as needed and take full responsibility for the content of the publication.

## Funding sources

This research did not receive any specific grant from funding agencies in the public, commercial, or not-for-profit sectors.

## CRediT authorship contribution statement

**Christoph Veigl:** Writing – review & editing, Writing – original draft, Methodology, Investigation, Formal analysis, Data curation, Conceptualization. **Jacqueline Eleonora Ek:** Writing – review & editing, Writing – original draft, Methodology, Formal analysis, Data curation. **Gavin D. Perkins:** Writing – review & editing, Writing – original draft, Supervision, Methodology, Investigation, Formal analysis, Data curation, Conceptualization.

## Declaration of competing interest

Christoph Veigl is member of the Young European Resuscitation Council (ERC) committee. Jacqueline Eleonora Ek is the Chair of the Diversity, Equity and Inclusion (DEI) Committee for the ERC and a member of the Young ERC. Gavin D. Perkins is Director of Science for the European Resuscitation Council and Editor in Chief of Resuscitation Plus. To ensure an independent review process, the author was not involved in the peer review, editorial decision-making, or acceptance of this manuscript. The views represented in this paper are the views of the authors and do not imply endorsement from affiliated professional societies.

## Appendix A Supplementary material

Supplement A

Supplement B Survey, used prompts for AI and detailed overview of the collected information.
