# Supplementary material for: Current European landscape of in-hospital cardiac arrest registries mapped by a cross-sectional survey including barriers and enablers setting up a registry
Source: Resusc Plus. 2026 Oct 16;32:101533. doi: 10.1016/j.resplu.2026.101533 (PMC13733453; doi:10.1016/j.resplu.2026.101533)
Supplement: Supplementary file 1 [file mmc1.docx]

**Survey on IHCA registries**

1) Which country/ NRC are you representing? (insert country name)
2) Approximate number of hospitals in your country (approximate number of hospitals):
3) What type of IHCA registry exists in your country? (multiple responses allowed)
   a) No registry
   b) National registry
   c) Regional registry
   d) Local registry
   e) Other (open ended answer)
4) Proportion of hospitals covered by registries: (%)
5) Population covered by registries? (multiple responses allowed)
   a) No information available
   b) Adults
   c) Children
   d) Neonates
6) Which cardiac arrests / cases are included in the registry? (one answer allowed)
   a) No information available
   b) All cardiac arrests
   c) Attended by hospital resuscitation team
   d) Cardiac arrests occurring in a particular part of the hospital (if ticked, also which one)
   e) other (open ended answer)
7) Which information is collected by the registries? (multiple responses allowed)
   a) No information available
   b) Incidence of IHCA
   c) Patient demographics
   d) Hospital name
   e) Location of cardiac arrest in the hospital
   f) Initial rhythm
   g) ROSC achieved
   h) Survival to discharge / 30 days
   i) Neurological outcome at discharge / 30 days
   j) Long term survival (> 30d)
   k) Long term neurological outcome (>30d)
   l) Other information (open ended answer)
8) If different types of registries (national, regional, local) collect different data, which are the substantial differences between them? (open end question)
9) URL for website for the registry (open ended question)

**Prompt used to perform thematic analysis with the help of Open-AI GPT5.3 on 28^th^ April, 2026:**

“Could you perform following task on the file I have attached: Qualitative data generated from the group discussions are analysed using a simple thematic approach: Review the notes from each group attached in the file, code key points into recurring themes, and group similar responses to identify common patterns across discussions.”
